# Unique Activity Spectrum of Colicin F_Y_: All 110 Characterized *Yersinia enterocolitica* Isolates Were Colicin F_Y_ Susceptible

**DOI:** 10.1371/journal.pone.0081829

**Published:** 2013-12-10

**Authors:** Juraj Bosák, Lenka Micenková, Martin Vrba, Alena Ševčíková, Daniela Dědičová, Debora Garzetti, David Šmajs

**Affiliations:** 1 Department of Biology, Faculty of Medicine, Masaryk University, Brno, Czech Republic; 2 Department of Clinical Microbiology, University Hospital Brno, Brno, Czech Republic; 3 National Reference Laboratory for *Salmonella*, The National Institute of Public Health, Prague, Czech Republic; 4 Max von Pettenkofer-Institute, Ludwig Maximilian University of Munich, Munich, Germany; Centre National de la Recherche Scientifique, Aix-Marseille Université, France

## Abstract

Colicin F_Y_ is a plasmid encoded toxin that recognizes a yersinia-specific outer membrane protein (YiuR) as a receptor molecule. We have previously shown that the activity spectrum of colicin F_Y_ comprises strains of the genus *Yersinia*. In this study, we analyzed the activity of colicin F_Y_ against 110 *Yersinia enterocolitica* isolates differing in geographical origin and source. All isolates were characterized through analysis of 16S rRNA genes, serotyping, biotyping, restriction profiling of genomic DNA, detection of virulence markers and susceptibility to antibiotics. This confirmed the broad variability of the collection, in which all 110 *Y. enterocolitica* isolates, representing 77 various strains, were inhibited by colicin F_Y_. Although isolates showed variable levels of susceptibility to colicin F_Y_, it was not associated with any strain characteristic. The universal susceptibility of *Y. enterocolitica* strains to colicin F_Y_ together with the absence of activity towards strains outside the *Yersinia* genus suggests potential therapeutic applications for colicin F_Y_.

## Introduction

Based on both genetic and phenotypic features, *Yersinia enterocolitica* is considered to be a heterogeneous species [1]–[3]. *Y. enterocolitica* is often found in aquatic environments and in various animal reservoirs, with swine being a major reservoir of human pathogenic strains. The most frequently isolated human strains belong to bioserotypes 1B/O:8, 2/O:5,27, 2/O:9, and 4/O:3, with 4/O:3 being the most common and typical for Europe [4]–[8].

Infections caused by *Y. enterocolitica* are the third most common bacterial alimentary infections of humans in the European Union [9]. Yersiniosis ranges from self-limited enteritis to life-threatening systemic infections. The most frequent manifestation is diarrhea, mainly affecting children [4], [10]–[14]. Although antibiotic treatment is recommended for serious cases, the benefits of antibiotic therapy in uncomplicated cases is not well established [5], [15]–[17]. Instead, rehydration and use of probiotics are often suggested for simple diarrheal cases.

Production of bacteriocins has been described in many genera of enteric bacteria including *Escherichia*, *Shigella*, *Citrobacter*, *Salmonella,* and *Yersinia* [18]. Although antibacterial activity of various species of genus *Yersinia* has been previously documented [19]–[24], only three yersinia-produced bacteriocins have been intensively studied and characterized on the molecular level. These include the bacteriocin of *Y. pestis* (pesticin I; [25]–[30]), a phage tail-like bacteriocin produced by *Y. enterocolitica* (enterocoliticin; [31], [32]), and a bacteriocin from *Y. frederiksenii* Y27601 (colicin F_Y_; [33]). Colicin F_Y_ is produced by an environmental isolate of *Y. frederiksenii*, which contains the colicinogenic plasmid pYF27601 (5,574 bp) harboring the colicin F_Y_ activity (*cfyA*) and immunity (*cfyI*) gene. Colicin F_Y_ (54 kDa) recognizes a yersinia-specific outer membrane protein (YiuR) as a receptor molecule in susceptible bacterial strains. YiuR protein is encoded by many yersiniae (e.g. *Y. pestis*, *Y. pseudotuberculosis*, *Y. enterocolitica*, and *Y. frederiksenii*). YiuR belongs to the family of TonB-dependent proteins with putative iron uptake function. Colicin F_Y_ uses the TonB system for translocation, similar to colicins B, D, Ia, and Ib. Lethal activity of colicin F_Y_ is exerted by formation of voltage-gated pore in the cytoplasmic membrane. The lethal effect of colicin F_Y_ is directed against several nonpathogenic and opportunistic yersiniae (i.e. *Y. frederiksenii*, *Y. aldovae*, *Y. kristensenii*, and *Y. intermedia*) and also against pathogenic strains of *Y. enterocolitica*. Previously published data [33] suggested that *Y. enterocolitica* is widely susceptible to colicin F_Y_; however, the strain collection was limited to only 31 *Y. enterocolitica* isolates originated in the Czech Republic that were not characterized in detail.

In this study, 110 *Y. enterocolitica* isolates with different geographical origins and sources were characterized in detail to exclude any potential clonal character of the isolates. Colicin F_Y_ inhibited growth of all tested isolates indicating that the vast majority of *Y. enterocolitica* strains are susceptible to colicin F_Y_.

## Materials and Methods

### Bacterial strains and growth conditions

Colicin F_Y_ producer, *Yersinia frederiksenii* strain Y27601, was obtained from the National Reference Laboratory for *Salmonella*, The National Institute of Public Health (NIPH), Prague. The recombinant strain producing colicin F_Y_ (*Escherichia coli* TOP10F'pDS1068) was constructed in our laboratory [33]. *Y. enterocolitica* isolates were obtained from several institutions including the National Reference Laboratory for *Salmonella*, NIPH, Prague; the Department of Clinical Microbiology, University Hospital Brno (UHB), Brno; and the Max von Pettenkofer-Institute (MvPI), Ludwig Maximilian University of Munich, Munich. Detailed information for the isolates of *Y. enterocolitica* used in this work is presented in Table S1. We used a set of 118 *Escherichia* isolates containing *E. coli* (39 isolates), *E. fergusonii* (10 isolates), *E. hermanii* (42 isolates), and *E. vulneris* (27 isolates). An additional 18 isolates of enterobacterial species included *Budvicia aquatica* (24510; from E. Aldová), *Citrobacter youngae* (42/57; NIPH), *C. braakii* (B718; UHB), *C. freundii* (B607; UHB), *Enterobacter aerogenes* (1832; NIPH), *E. cloacae* (B604; UHB), *Klebsiella pneumoniae* (B615; UHB), *K. oxytoca* (B632; UHB), *Kluywera ascorbata* (B792; UHB), *Leclercia adecarboxylata* (2666; from I. Sedláček), *Morganella morganii* (B619; UHB), *Pragia fontium* (24613; from E. Aldová), *Proteus vulgaris* (B635; UHB), *Serratia ficaria* (B779; UHB), *Salmonella enterica subsp. enterica* (B753; UHB), *Shigella flexneri* (strain 4; [66]), *S. sonnei* (strain 17; from laboratory stock), and *S. boydii* (U1; from V. Horák).

Tryptone-yeast (TY) broth consisting of 8 g/l tryptone (Hi-Media, Mumbai, India), 5 g/l yeast extract (Hi-Media), and 5 g/l sodium chloride in water was used throughout the study. For cultivation on solid media, TY broth was supplemented with agar powder (1.5%, w/v; Hi-Media). Mueller-Hinton agar (38 g/l; Hi-Media) was used for analysis of antibiotic susceptibility. TY agar plates supplemented with L-(+)-arabinose (0.2 g/l; Sigma-Aldrich, St. Louis, USA) were used to induce expression of colicin F_Y_ in recombinant *E. coli* TOP10F'pDS1068.

### 16S rRNA analysis

To analyze the 16S rDNA in *Y. enterocolitica* isolates, a part of the 16S rRNA gene, consisting of 524 bp, was amplified from a single bacterial colony resuspended in 100 µl of deionized water. The yersinia DNA was amplified using *Taq* polymerase (New England Biolabs, Beverly, USA) and a pair of 16S rDNA-specific primers (16SRNA-F: 5′-AGTTTGATCATGGCTCAG-3′ and 16SRNA-R: 5′-TTACCGCGGCTGCTGGCA-3′) [34]. Colony PCR started with denaturation at 94°C for 5 min, followed by 40 cycles at 95°C for 30 s, 50°C for 30 s, 72°C for 1 min, and extension at 72°C for 10 min. PCR products were sequenced using *Taq* DyeDeoxy Terminator Cycle Sequencing Kit (Applied Biosystems, Foster City, USA). The Lasergene program package (DNASTAR, Madison, USA) was used for assembly of the sequencing reads and further data analyses. Isolates were classified to subspecies on the basis of polymorphisms in the 30 bp region of the 16S rDNA [35].

### Bioserotype classification

*Y. enterocolitica* isolates were serotyped and biotyped using previously described methods [1], [2]. Isolates from the Czech Republic were serotyped using diagnostic agglutination antisera O:3, O:5, O:8 and O:9 (ITEST PLUS, Hradec Králové, Czech Republic) and biotyped on the basis of esculin hydrolysis, indole production, xylose and/or trehalose utilization. Bioserotypes of 50 isolates from outside the Czech Republic were provided with isolates. For verification of the provided bioserotype characterization, a random subgroup (n = 15) of these isolates was also bioserotyped.

### Pulsed field gel electrophoresis (PFGE)

Overnight TY cultures were centrifuged, diluted in suspension buffer (100 mM Tris (Sigma-Aldrich), 100 mM EDTA (Sigma-Aldrich), pH = 8) to OD_600_ = 1.4 and mixed with equal volume of 1.6% Pulsed Field Certified Agarose (Bio-Rad Laboratories, Hercules, USA) containing 1% SDS (Sigma-Aldrich). Proteinase K (Sigma-Aldrich) was added to the suspension (to a final concentration of 0.5 mg/ml) and samples were aliquoted into plug molds. Each plug was transferred into 5 ml of lysis buffer (50 mM Tris, 50 mM EDTA, 1% SDS, 500 µg proteinase K, pH = 8) and incubated at 54°C for 2 hours. The plugs were then washed in deionized water at 54°C (2×15 min) followed by washing (4×15 min) with TE buffer (10 mM Tris, 1 mM EDTA, pH = 8). After bacterial lysis, the plugs were digested with 50 U of *Not*I enzyme (New England Biolabs) at 37°C for 3 hours and were loaded into a 1% Pulsed Field Certified Agarose gel. Electrophoresis was performed using a CHEF-DR II system (Bio-Rad Laboratories) in 0.5% TBE (50 mM Tris, 50 mM boric acid, 1.5 mM EDTA) at 14°C, 6 V/cm and a ramping time of 2.5 s to 25 s over 24 hours. Gels were stained with ethidium bromide (1 µg/ml) and visualized under UV light. Genomic DNA from *Salmonella enterica*, serotype Braenderup H9812, digested using *Xba*I enzyme (New England Biolabs), was used as a molecular weight standard.

BioNumerics v6.1 (Applied Maths, Sint Martens Latem, Belgium) was used to analyze restriction profiles (bands from ∼50 kbp to ∼500 kbp). Based on the PFGE data, dendrograms were constructed using Dice's coefficient of similarity and Unweighted Pair Group Method with Arithmetic Mean (UPGMA) clustering at 0.5% tolerance.

### Detection of virulence factors

Genomic DNA was isolated from overnight cultures using DNAzol® Reagent (Invitrogen, Carlsbad, USA), according to the manufacturer's instructions. Isolated DNA (1 µl) was used as a template for multiplex PCR [36]. Three different virulence markers – *ystA* (gene encoding yersinia stable toxin, 134 bp), *virF* (gene encoding transcriptional activator of *yop* genes, 231 bp) and *ail* (attachment and invasion locus, 356 bp), were amplified using 0.5 U *Taq* polymerase (New England Biolabs) and specific primer pairs (Yst-a: 5′-GTCTTCATTTGGAGGATTCGGC-3′, Yst-b: 5′-AATCACTACTGACTTCGGCTGG-3′, ViF-a: 5′-GCTTTTGCTTGCCTTTAGCTCG-3′, VirF-b: 5′-AGAATACGTCGCTCGCTTATCC-3′, Ail-a: 5′-TGGTTATGCGCAAAGCCATGT-3′, and Ail-b: 5′-TGGAAGTGGGTTGAATTGCA-3′).

PCR started with denaturation at 94°C for 5 min, followed by 35 cycles at 94°C for 30 s, 55°C for 30 s, 72°C for 1 min, and extension at 72°C for 10 min. When multiplex PCR results were negative, virulence markers were analyzed separately under the same PCR conditions.

### Antibiotic susceptibility assay

Yersiniae were tested for susceptibility to 14 antibiotics using the disc diffusion method and the National Committee for Clinical Laboratory Standards guidelines [37]. Susceptibility assays with antibiotic disks (Oxoid, Basingstoke, UK) were performed on Mueller–Hinton agar at 37°C. The following antimicrobial drugs and quantities were used: ampicillin (AMP; 10 mg), cephalothin (KF; 30 mg), doxycycline (DO; 30 mg), cefuroxime (CXM; 30 mg), ciprofloxacin (CIP; 5 mg), sulfamethoxazole-trimethoprim (SXT; 25 mg), oxolinic acid (OA; 30 mg), gentamicin (CN; 10 mg), cefotaxime (CTX; 30 mg), ceftazidime (CAZ; 30 mg), amoxicillin with clavulanic acid (AMC; 30 mg), aztreonam (ATM; 30 mg), chloramphenicol (C; 30 mg), and colistin sulphate (CT; 10 mg).

### Colicin activity assay

Detection of colicin activity was performed as described previously [38]. Briefly, the agar plates were inoculated with a stab from the bacterial culture (*Y. frederiksenii* Y27601, *E. coli* TOP10F' carrying pDS1068, and *E. coli* TOP10F' carrying pBAD-A), incubated at 37°C for 48 hours and the resulting macrocolonies were killed with chloroform vapors. Each plate was overlaid with a 0.75% TY agar (3 ml) containing 1×10^8^ cells of a tested isolate. Simultaneously, the level of bacterial susceptibility to colicin F_Y_ was detected by spotting of serial dilutions (diluting factor 0.25) of purified colicin F_Y_ [33] on the same agar plates. The plates were then incubated at 25°C overnight and zones of growth inhibition were read. The reciprocal value of the highest dilution of the purified colicin causing complete and partial growth inhibition (clear and turbid zone, respectively) of susceptible bacteria was considered to be the colicin titer (in arbitrary units, A.U.).

### Detection of colicin F_Y_ immunity gene

To analyze for the presence of the colicin F_Y_ immunity gene in *Y. enterocolitica* isolates, a part of the *cfyI* gene, consisting of 162 bp, was amplified from a single bacterial colony resuspended in 100 µl of deionized water. Bacterial suspensions (1 µl) were boiled and used as a DNA templates, which were amplified using *Taq* polymerase (New England Biolabs) and specific primers (immFy-F: 5′-GGACGTTACCGCCTACGG-3′ and immFy-R: 5′-ACCCTGAAAGCGAACGAC-3′). Colony PCR started with denaturation at 94°C for 5 min, which was followed by 40 cycles at 95°C for 30 s, 58°C for 30 s, 72°C for 1 min, and finished by extension at 72°C for 10 min.

### Sequencing of *yiu*R and *ton*B genes

The *yiuR* gene from nine *Y. enterocolitica* isolates (YE48, YE61, YE66, YE73 YE81,YE84, YE85, YE93, and YE97) was amplified from a single colony using *Taq* polymerase (New England Biolabs) and specific primers (YE1461SD-F: 5′-ACCGAAATAAATGGCTAAGGCCTTTAGG-3′, and YE1461-R: 5′-TTAGAAATCGTAGCTGGCGC-3′). Colony PCR started with denaturation at 94°C for 5 min, followed by 40 cycles at 95°C for 30 s, 58°C for 2 min, 72°C for 1 min, and extension at 72°C for 10 min. Additionally, the *tonB* gene was amplified using specific primers (tonB_YE2222-F: 5′-ATGCAGCTAAATAAATTTTTCTTGGG-3′ and tonB_YE2222-R: 5′-TTAGTCCATTTCCGTCGTG-3′). All PCR products were sequenced using a *Taq* DyeDeoxy Terminator Cycle Sequencing Kit (Applied Biosystems). The Lasergene program package (DNASTAR) was used for assembly of the sequencing reads and further data analyses.

## Results

### Sequencing of 16S rRNA genes, serological and biochemical characterization of isolates

Sequencing analysis of the 16S rRNA coding DNA confirmed that all 110 tested isolates belonged to the *Y. enterocolitica* species (data not shown). Moreover, sequencing results were used to classify isolates into subspecies; 108 isolates were identified as *Y. enterocolitica* subsp. *palearctica* and 2 isolates as *Y. enterocolitica* subsp. *enterocolitica* (Fig. S1). Seven different serotypes and all six different biotypes (1A, 1B, 2, 3, 4, and 5) were present within the isolates (Fig. S1). Common human pathogenic serotypes, (i.e. O:3, O:5,27, O:8, and O:9) and also several atypical serotypes (i.e. O:5, O:6,30 and O:36) were present in our collection. *Y. enterocolitica* bioserotype 4/O:3 was the most frequent (77%).

### PFGE analysis

**Figure 1:**
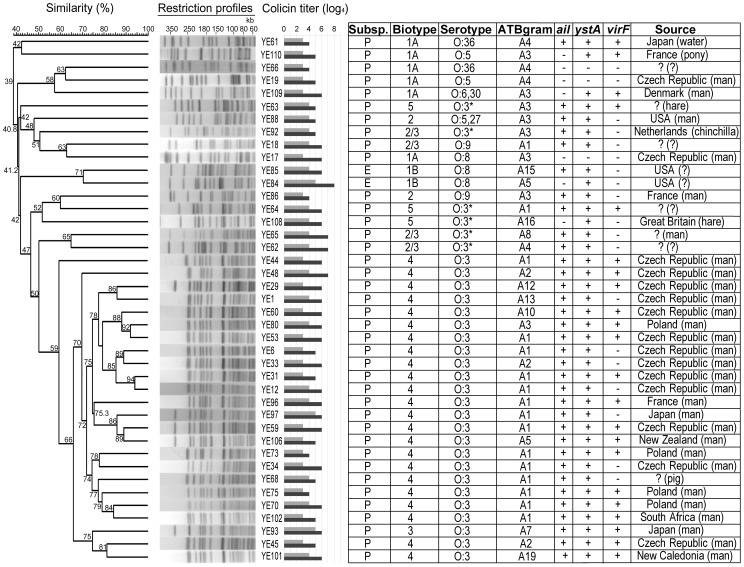
Identified pulsotypes among *Y. enterocolitica* isolates. Similarities (%) between restriction patterns were calculated using the Dice's index and are shown as the numbers close to nodes. The data were sorted using the UPGMA method. For construction of this dendrogram, 41 *Y. enterocolitica* pulsotypes with similarity lower than 85% were selected from a dendrogram containing all 110 isolates (Fig. S1). Susceptibility to colicin F_Y_ is shown in the right panel, followed by additional strain characteristics. Colicin F_Y_ titers are shown as the reciprocal exponent of the highest four-fold dilution causing clear (light grey) and turbid (dark grey) zones of inhibition. Subspecies: P =  *palearctica*, E =  *enterocolitica*. *Serotypes O:1 and O:2 have been combined to O:3 serotype according to [1].

To determine the genetic variability of *Y. enterocolitica* isolates, restriction profiles of all isolates were determined (Fig. S1). Altogether, 41 various pulsotypes (at the 85% similarity level) were identified (Fig. 1). Moreover, 24 different pulsotypes were found within the most abundant bioserotype 4/O:3 subgroup.

### Identification of virulence markers

Two chromosome- and one plasmid-encoded virulence markers (*ail*, *ystA*, and *virF*, respectively) were detected using PCR (Fig. S1). In contrast to the chromosomal genes, which were detected in more than 90% of isolates (91% and 96% for *ail* and *ystA*, respectively), the *virF* gene was found in only 50% of *Y. enterocolitica* isolates. All three determinants were identified in 52 isolates (47%) and only four isolates were negative for all tested markers. Taken together, most of the collected isolates could be considered to be potentially pathogenic *Y. enterocolitica*.

### Susceptibility to antibiotics

All tested yersiniae were susceptible to ciprofloxacin, cefotaxime, ceftazidime, aztreonam, and colistin sulphate. About 20% of isolates showed an intermediate susceptibility to cefuroxime and amoxicillin with clavulanic acid. In addition, more than 90% of isolates were susceptible to doxycycline, sulfamethoxazole-trimethoprim, oxolinic acid, gentamicin, and chloramphenicol. Less than 10% of yersiniae were susceptible to ampicillin and cephalothin. Altogether, 20 different antibiograms were identified (Table S2), with the most frequent antibiogram, A1, identified in 46% of isolates (Fig. S1). Isolates with antibiogram A1 were susceptible to all tested antibiotics with the exception of ampicillin and cephalothin.

### Susceptibility to colicin F_Y_

**Figure 2:**
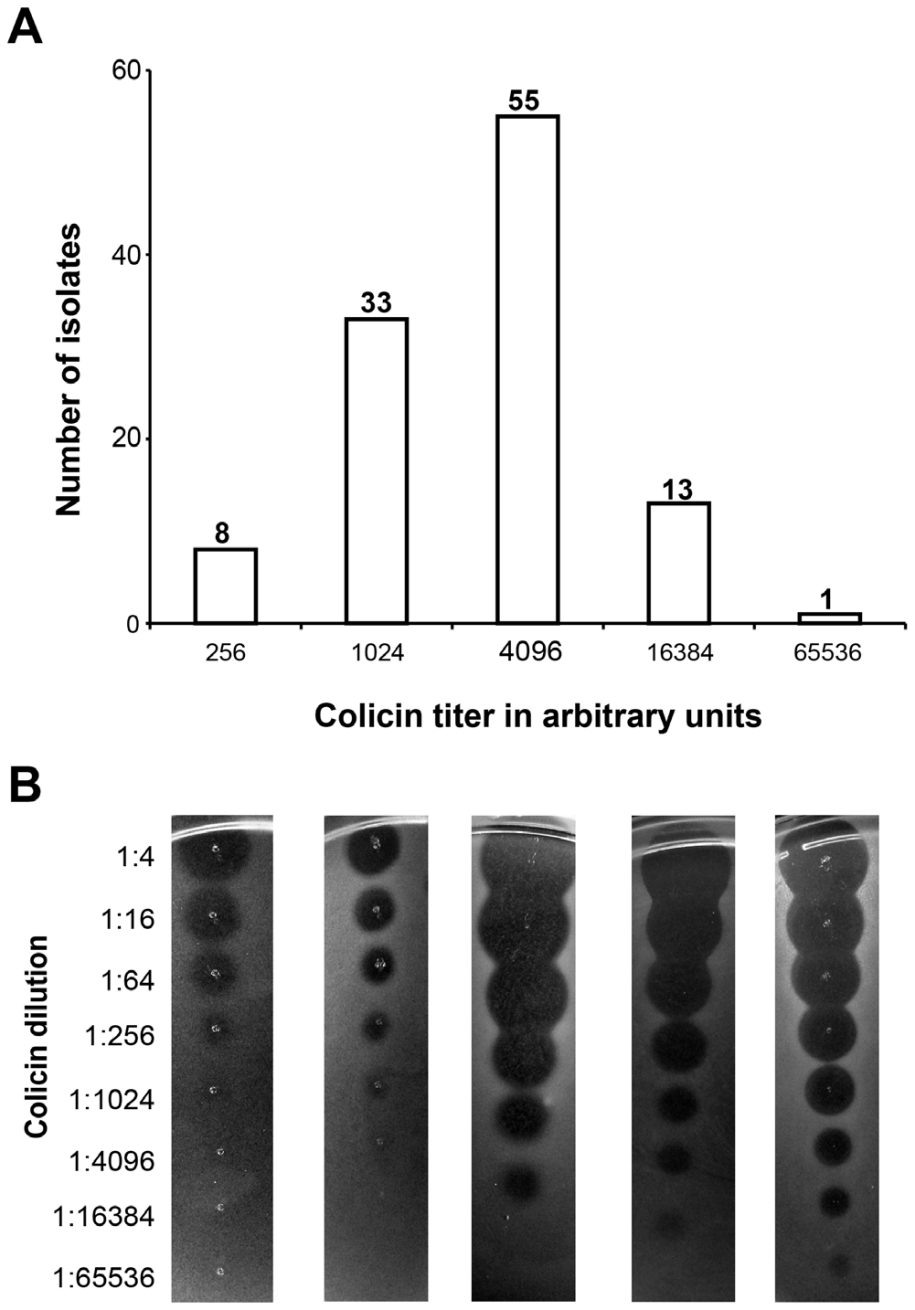
Susceptibility of *Y. enterocolitica* isolates to colicin F_Y_. A) Distribution of colicin F_Y_ susceptibility levels. Colicin F_Y_ susceptibility is shown as colicin titers representing the highest dilution causing detectable growth inhibition (last turbid zone). The susceptibility to colicin F_Y_ ranged from 256 to 65536 A.U. Half of the *Y. enterocolitica* isolates showed susceptibility corresponding to titer of 4096 A.U. B) Inhibition zones corresponding to different levels of susceptibility. Isolates YE75, YE24, YE107, YE99, and YE84 were used as representative indicators.

**Figure 3:**
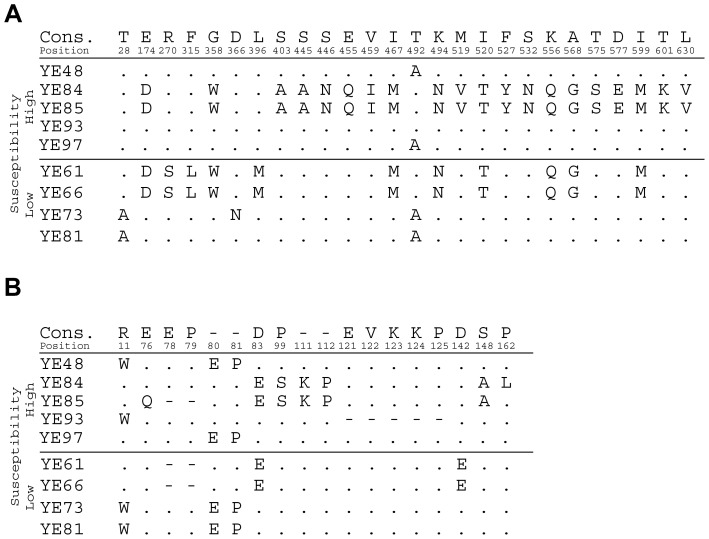
Sequence variability between highly and less susceptible *Y. enterocolitica* isolates. A) Sequence analysis of YiuR protein from various isolates identified variability in 26 amino acid posistions. B) Sequence analysis of TonB from various isolates identified variability in 18 amino acid positions. A consensus sequence is shown at the top of the figure. The numbers correspond to amino acid position in the YiuR and TonB proteins, respectively.

Colicin F_Y_ producers, *Y. frederiksenii* Y27601 and a recombinant strain of *E. coli* carrying pDS1068 [33], inhibited growth of all tested *Y. enterocolitica* isolates. All *Y. enterocolitica* isolates were also susceptible to purified colicin F_Y_. Degree of susceptibility to purified colicin F_Y_ (shown as a colicin titer in A.U.) varied from 64 to 1024 and from 256 to 65536 for clear and turbid zones of growth inhibition, respectively. The susceptibility of individual isolates of *Y. enterocolitica* to colicin F_Y_ is shown in Fig. S1 and the distribution of colicin F_Y_ susceptibility among *Y. enterocolitica* is shown in Fig. 2. The susceptibility of *Y. enterocolitica* isolates to colicin F_Y_ showed a nonrandom distribution (chi-squared goodness of fit test; p≤0.001), where the majority of isolates were susceptible to 4096 A.U. for turbid zones of growth inhibition. Sequence analysis of two proteins (YiuR and TonB) in five highly and four less susceptible isolates identified variability within isolates (26 and 18 amino acid positions, respectively). However, not a single variable position of YiuR or TonB was associated with the difference in susceptibility to colicin F_Y_ (Fig. 3). All 110 isolates did not encode a colicin F_Y_ immunity protein, which should affect the susceptibility to colicin F_Y_.

In contrast to the universal susceptibility of *Y. enterocolitica* strains to colicin F_Y_, no colicin F_Y_-susceptible strains outside the *Yersinia* genus were identified when 136 isolates belonging to the 13 other enterobacterial genera (i.e. *Budvicia, Citrobacter*, *Enterobacter, Escherichia*, *Klebsiella, Kluyvera, Leclercia, Morganella, Pragia, Proteus*, *Salmonella*, *Serratia*, and *Shigella*) were tested (data not shown).

## Discussion

The aim of this study was to evaluate susceptibility of *Y. enterocolitica* to colicin F_Y_ using a set of characterized isolates. The 110 isolates were collected from different geographical areas with the majority of isolates originating in Europe. Most of *Y. enterocolitica* isolates originated from human clinical material, but veterinary and environmental isolates were also present. To exclude the possibility that the collection of *Y. enterocolitica* contains multiple isolates of identical strains, further characterization of these isolates was performed including analysis of the 16S rRNA genes, serotyping, biotyping, restriction profiling of genomic DNA, detection of virulence markers and susceptibility to antibiotics. Using these typing techniques, 77 different *Y. enterocolitica* strains were identified in our collection, from which 59 strains were represented by a single isolate, while the other 18 strains contained more than one isolate with identical characteristics. High genetic variability was shown for nonhuman isolates, while more subtle variability was found within the 4/O:3 subgroup. Almost all tested isolates can be considered to be pathogenic or potentially pathogenic. With the exception of two beta-lactam antibiotics, isolates of *Y. enterocolitica* were susceptible to all tested antibiotics. As results of the characterization assays are in agreement with other studies on *Y. enterocolitica* [5], [9], [39]–[48], our set of *Y. enterocolitica* isolates represents a group of isolates with typical features.

None of the110 isolates of *Y. enterocolitica* encoded the colicin F_Y_ immunity protein (data not shown) and all were susceptible to colicin F_Y_; however they differed in the degree of susceptibility to it. No obvious association between colicin F_Y_ susceptibility and other described strain parameters was found, indicating that it is independent of other strains characteristics (Fig. S1). Moreover, no amino acid replacements in the YiuR and TonB proteins were associated with differences in colicin F_Y_ susceptibility. Others features (e.g. cell wall composition) seem to affect susceptibility to colicin F_Y_.

Bacterial resistance to bacteriocins is considered to be a successful strategy in antimicrobial competition [49]–[51]. It has been shown that around 75% of *E. coli* strains isolated from different source populations are resistant to one or more bacteriocin types [52], [53]. Resistance to bacteriocin-like substances produced by yersiniae has been found among *Y. enterocolitica* strains [21]. In addition, enterocoliticin, a characterized phage tail-like bacteriocin, has been shown to inhibit pathogenic *Y. enterocolitica* serotypes O:3, O:5,27, and O:9, but not serotype O:8 and various nonpathogenic isolates (biotype 1A) [31]. In contrast, colicin F_Y_ appears to inhibit all *Y. enterocolitica* isolates. A similar finding has been published with respect to an uncharacterized bacteriocin from *Y. kristensenii* and 35 tested strains of *Y. enterocolitica* [24], [54], [55]; however, it is not known whether this uncharacterized bacteriocin could, in fact, be colicin F_Y_.

Although the reason for the universal susceptibility of *Y. enterocolitica* to colicin F_Y_ is unknown, it can be speculated that this could be a result of the clonal character of isolates and/or the essential character of the YiuR receptor for yersiniae. The clonal character of *Y. enterocolitica* was obvious only in pathogenic isolates (e.g. serotype O:3), while nonpathogenic isolates of biotype 1A were more heterogeneous [56], [57]. This explanation therefore appears unlikely. Similarly, the essential character of the YiuR receptor also appears unlikely, since yersiniae possess several iron uptake systems (e.g. *Y. pestis* harbors several systems and Yiu-mediated transport is not first in the iron uptake hierarchy [58]). Another possible explanation for universal activity of colicin F_Y_ against *Y. enterocolitica* involves a close relationship between *Y. enterocolitica* and colicin F_Y_ producers, with little or no contact between them. A phylogenetic analysis showed that *Y. enterocolitica* was clearly related to other environmental enterocolitica-like species including *Y. frederiksenii* [59], [60]. In addition, contact between the two bacterial species appears to be quite rare despite the sporadic co-occurrence of both species [61]–[63]. The separation of *Y. enterocolitica* strains from environmental colicin F_Y_-producers could explain the absence of colicin F_Y_ resistant mutants among *Y. enterocolitica* strains. In fact, colicin F_Y_ resistant colonies of *Y. enterocolitica* were induced in the presence of colicin F_Y_ in laboratory conditions (data not shown). Moreover, the environmental yersiniae (e.g. *Y. frederiksenii* and *Y. kristensenii*) that live in the same environment as colicin producers contain both resistant and susceptible strains to colicin F_Y_ [33].

There is increasing interest in nonpathogenic microorganisms and their antimicrobial substances that naturally antagonize pathogenic agents. To date, several nonpathogenic *Escherichia coli* strains have been used as probiotics (e.g. *E. coli* Nissle 1917; [64]). Although the role of bacteriocin synthesis in probiotic bacteria is not known, production of bacteriocins is a common feature of many of them [65]–[67]. It is therefore tempting to speculate that synthesis of colicin F_Y_ could represent an important feature of recombinant probiotic *E. coli* strains used in cases of diarrhea caused by yersiniae. However, the effect of colicin F_Y_ synthesis should be tested using *in vivo* experiments to see whether colicin F_Y_ has therapeutic potential relative to intestinal yersiniosis.

## Supporting Information

Figure S1 **Dendrogram of** ***Y. enterocolitica*** **isolates.** Similarities (%) between restriction patterns were calculated using the Dice's index and are shown as the numbers close to nodes. The data were sorted using the UPGMA method. Susceptibility to colicin F_Y_ is shown in the right panel, followed by additional strain characteristics. Colicin F_Y_ titers are shown as the reciprocal exponent of the highest four-fold dilution causing clear (light grey) and turbid (dark grey) zones of inhibition. *Serotypes O:1 and O:2 have been combined to O:3 serotype according to [1]. The lines on the right side show isolates with the same characteristics (i.e. considered to be identical strains).
(TIF)

Table S1 *Y. enterocolitica* isolates used in this study.
(DOCX)

Table S2 Antibiotic susceptibility of *Y. enterocolitica* isolates.
(DOCX)
