# Supplementary figures and images for: Unique Activity Spectrum of Colicin FY: All 110 Characterized Yersinia enterocolitica Isolates Were Colicin FY Susceptible
Source: PLoS One. 2013 Dec 10;8(12):e81829. doi: 10.1371/journal.pone.0081829 (PMC3858286; doi:10.1371/journal.pone.0081829)

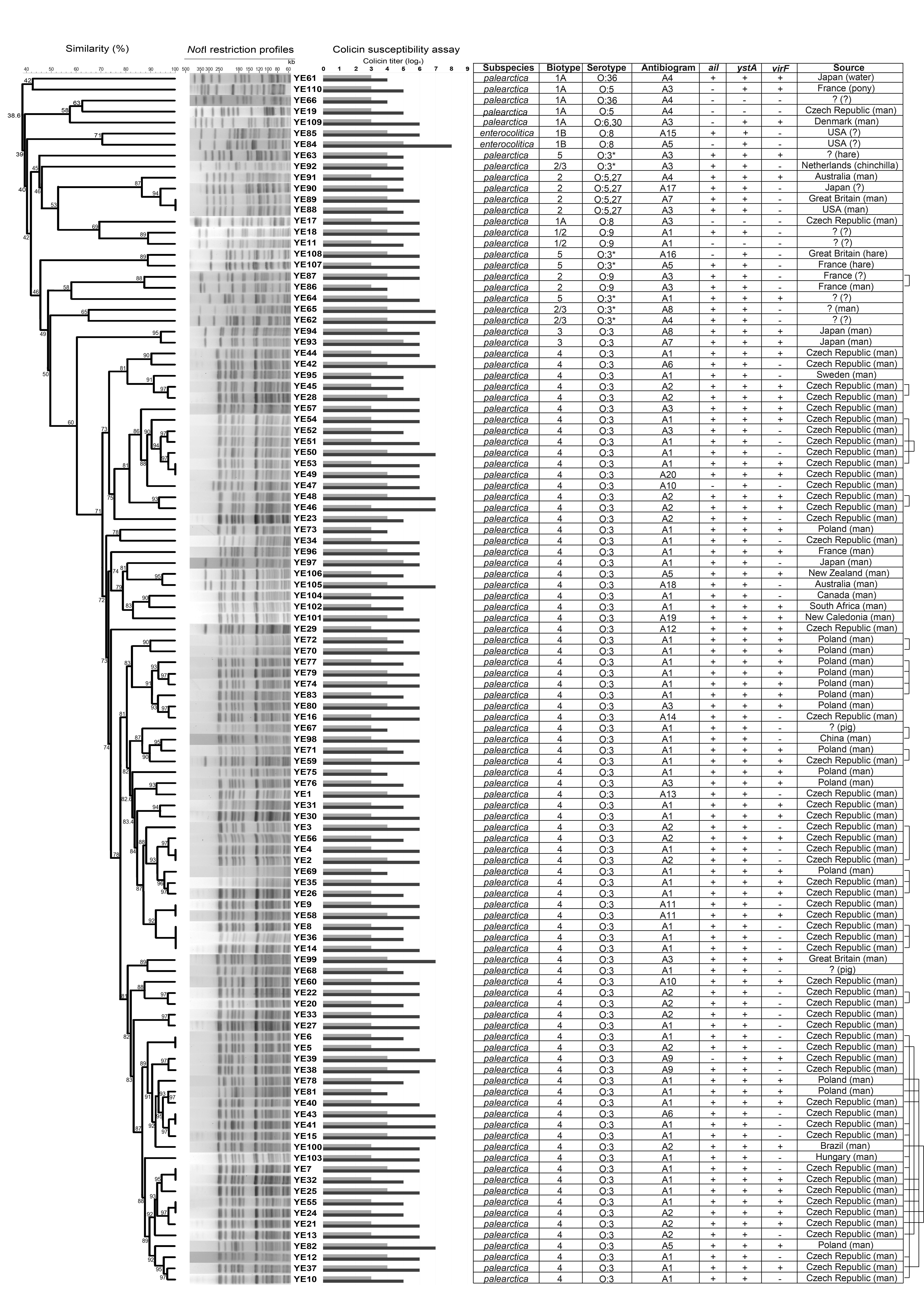

Supplement: Figure S1 — Dendrogram of Y. enterocolitica isolates. Similarities (%) between restriction patterns were calculated using the Dice's index and are shown as the numbers close to nodes. The data were sorted using the UPGMA method. Susceptibility to colicin FY is shown in the right panel, followed by additional strain characteristics. Colicin FY titers are shown as the reciprocal exponent of the highest four-fold dilution causing clear (light grey) and turbid (dark grey) zones of inhibition. *Serotypes O:1 and O:2 have been combined to O:3 serotype according to [1]. The lines on the right side show isolates with the same characteristics (i.e. considered to be identical strains). (TIF) [file pone.0081829.s001.tif]
