# Supplementary material for: Unique Activity Spectrum of Colicin FY: All 110 Characterized Yersinia enterocolitica Isolates Were Colicin FY Susceptible
Source: PLoS One. 2013 Dec 10;8(12):e81829. doi: 10.1371/journal.pone.0081829 (PMC3858286; doi:10.1371/journal.pone.0081829)
Supplement: Table S1 — Y. enterocolitica isolates used in this study. (DOCX) [file pone.0081829.s002.docx]

**Table S1:** ***Y. enterocolitica* isolates used in this study.**

| **Isolate** | **Geographical origin** | **Source** | **Note *** |
| --- | --- | --- | --- |
| YE 1 | Czech Republic | human | 5Ye03 (NIPH) |
| YE 2 | Czech Republic | human | 15Ye03 (NIPH) |
| YE 3 | Czech Republic | human | 1Ye06 (NIPH) |
| YE 4 | Czech Republic | human | 3Ye07 (NIPH) |
| YE 5 | Czech Republic | human | 6Ye07 (NIPH) |
| YE 6 | Czech Republic | human | 7Ye07 (NIPH) |
| YE 7 | Czech Republic | human | 1Ye08 (NIPH) |
| YE 8 | Czech Republic | human | 2Ye08 (NIPH) |
| YE 9 | Czech Republic | human | 3Ye08 (NIPH) |
| YE 10 | Czech Republic | human | 7Ye08 (NIPH) |
| YE 11 | unknown | unknown | - (NIPH) |
| YE 12 | Czech Republic | human | 1Ye10 (NIPH) |
| YE 13 | Czech Republic | human | 4Ye09 (NIPH) |
| YE 14 | Czech Republic | human | 7Ye09 (NIPH) |
| YE 15 | Czech Republic | human | 8Ye08 (NIPH) |
| YE 16 | Czech Republic | human | 7Ye06 (NIPH) |
| YE 17 | Czech Republic | human | 5Ye06 (NIPH) |
| YE 18 | unknown | unknown | - (NIPH) |
| YE 19 | Czech Republic | human | 1Ye03 (NIPH) |
| YE 20 | Czech Republic | human | 7578 (UHB) |
| YE 21 | Czech Republic | human | 7782 (UHB) |
| YE 22 | Czech Republic | human | 8008 (UHB) |
| YE 23 | Czech Republic | human | 7886 (UHB) |
| YE 24 | Czech Republic | human | 8472 (UHB) |
| YE 25 | Czech Republic | human | 8773 (UHB) |
| YE 26 | Czech Republic | human | 8703 (UHB) |
| YE 27 | Czech Republic | human | 8886 (UHB) |
| YE 28 | Czech Republic | human | 9081 (UHB) |
| YE 29 | Czech Republic | human | 9102 (UHB) |
| YE 30 | Czech Republic | human | 9464 (UHB) |
| YE 31 | Czech Republic | human | 10141 (UHB) |
| YE 32 | Czech Republic | human | 9953 (UHB) |
| YE 33 | Czech Republic | human | 9949 (UHB) |
| YE 34 | Czech Republic | human | 2209 (UHB) |
| YE 35 | Czech Republic | human | 3033 (UHB) |
| YE 36 | Czech Republic | human | 3316 (UHB) |
| YE 37 | Czech Republic | human | 7392 (UHB) |
| YE 38 | Czech Republic | human | 7250 (UHB) |
| YE 39 | Czech Republic | human | 4749 (UHB) |
| YE 40 | Czech Republic | human | 5258 (UHB) |
| YE 41 | Czech Republic | human | 4466 (UHB) |
| YE 42 | Czech Republic | human | 6050 (UHB) |
| YE 43 | Czech Republic | human | 7668 (UHB) |
| YE 44 | Czech Republic | human | 7563 (UHB) |
| YE 45 | Czech Republic | human | 7731 (UHB) |
| YE 46 | Czech Republic | human | 7852 (UHB) |
| YE 47 | Czech Republic | human | 9105 (UHB) |
| YE 48 | Czech Republic | human | 7852b (UHB) |
| YE 49 | Czech Republic | human | 9400 (UHB) |
| YE 50 | Czech Republic | human | 146 (UHB) |
| YE 51 | Czech Republic | human | 8523 (UHB) |
| YE 52 | Czech Republic | human | 8381 (UHB) |
| YE 53 | Czech Republic | human | 823 (UHB) |
| YE 54 | Czech Republic | human | 1541 (UHB) |
| YE 55 | Czech Republic | human | 3504 (UHB) |
| YE 56 | Czech Republic | human | 4364 (UHB) |
| YE 57 | Czech Republic | human | 7825 (UHB) |
| YE 58 | Czech Republic | human | 8264 (UHB) |
| YE 59 | Czech Republic | human | 8282 (UHB) |
| YE 60 | Czech Republic | human | 9375 (UHB) |
| YE 61 | Japan | water | IP2222 (MvPI) |
| YE 62 | unknown | unknown | gk132 (MvPI) |
| YE 63 | unknown | hare | gk1142 (MvPI) |
| YE 64 | unknown | unknown | gk2943 (MvPI) |
| YE 65 | unknown | human | JDE029 (MvPI) |
| YE 66 | unknown | unknown | Y101 (MvPI) |
| YE 67 | unknown | pig | Y141 (MvPI) |
| YE 68 | unknown | pig | Y142 (MvPI) |
| YE 69 | Poland | human | 40/97 (MvPI) |
| YE 70 | Poland | human | 146/97 (MvPI) |
| YE 71 | Poland | human | 241/97 (MvPI) |
| YE 72 | Poland | human | 120/98 (MvPI) |
| YE 73 | Poland | human | 683/98 (MvPI) |
| YE 74 | Poland | human | 910/98 (MvPI) |
| YE 75 | Poland | human | 120/99 (MvPI) |
| YE 76 | Poland | human | 128/99 (MvPI) |
| YE 77 | Poland | human | 627/99 (MvPI) |
| YE 78 | Poland | human | 99/96 (MvPI) |
| YE 79 | Poland | human | 243/96 (MvPI) |
| YE 80 | Poland | human | 252/96 (MvPI) |
| YE 81 | Poland | human | 353/96 (MvPI) |
| YE 82 | Poland | human | 159/97 (MvPI) |
| YE 83 | Poland | human | 184/97 (MvPI) |
| YE 84 | USA | unknown | IP636 (MvPI) |
| YE 85 | USA | unknown | IP19049(MvPI) |
| YE 86 | France | human | IP22393 (MvPI) |
| YE 87 | France | unknown | IP22394 (MvPI) |
| YE 88 | USA | human | IP199 (MvPI) |
| YE 89 | Great Britain | human | IP885 (MvPI) |
| YE 90 | Japan | unknown | IP1607 (MvPI) |
| YE 91 | Australia | human | IP22460 (MvPI) |
| YE 92 | Netherlands | chinchilla | IP135 (MvPI) |
| YE 93 | Japan | human | IP24231 (MvPI) |
| YE 94 | Japan | human | IP24232 (MvPI) |
| YE 95 | Sweden | human | Y244 (MvPI) |
| YE 96 | France | human | IP21981 (MvPI) |
| YE 97 | Japan | human | IP1601 (MvPI) |
| YE 98 | China | human | IP19718 (MvPI) |
| YE 99 | Great Britain | human | IP23222 (MvPI) |
| YE 100 | Brazil | human | IP23357 (MvPI) |
| YE 101 | New Caledonia | human | IP24309 (MvPI) |
| YE 102 | South Africa | human | IP7032 (MvPI) |
| YE 103 | Hungary | human | IP3692 (MvPI) |
| YE 104 | Canada | human | IP4115 (MvPI) |
| YE 105 | Australia | human | IP25728 (MvPI) |
| YE 106 | New Zealand | human | IP23230 (MvPI) |
| YE 107 | France | hare | IP1 (MvPI) |
| YE 108 | Great Britain | hare | IP178 (MvPI) |
| YE 109 | Denmark | human | IP102 (MvPI) |
| YE 110 | France | pony | IP124 (MvPI) |

*Original designation of the isolate. Institution providing the isolate is shown in parenthesis - NIPH (The National Institute of Public Health), UHB (University Hospital Brno), and MvPI (Max von Pettenkofer-Institute).
