# Supplementary material for: Unique Activity Spectrum of Colicin FY: All 110 Characterized Yersinia enterocolitica Isolates Were Colicin FY Susceptible
Source: PLoS One. 2013 Dec 10;8(12):e81829. doi: 10.1371/journal.pone.0081829 (PMC3858286; doi:10.1371/journal.pone.0081829)
Supplement: Table S2 — Antibiotic susceptibility of Y. enterocolitica isolates. (DOCX) [file pone.0081829.s003.docx]

**Table S2.** **Antibiotic susceptibility of *Y. enterocolitica* isolates.**

| **Antibiogram** | **Resistance** | **Intermediate susceptibility** | **Susceptibility** | **No. of isolates** |
| --- | --- | --- | --- | --- |
| **A1** | AMP, KF | - | DO, CXM, CIP, SXT, OA, CN, CTX, CAZ, AMC, ATM, C, CT | 51 |
| **A2** | AMP, KF | CXM | DO, CIP, SXT, OA, CN, CTX, CAZ, AMC, ATM, C, CT | 16 |
| **A3** | AMP, KF | AMC | DO, CXM, CIP, SXT, OA, CN, CTX, CAZ, ATM, C, CT | 13 |
| **A4** | AMP, KF, AMC | - | DO, CXM, CIP, SXT, OA, CN, CTX, CAZ, ATM, C, CT | 5 |
| **A5** | AMP | KF | DO, CXM, CIP, SXT, OA, CN, CTX, CAZ, AMC, ATM, C, CT | 4 |
| **A6** | AMP, KF, DO | - | CXM, CIP, SXT, OA, CN, CTX, CAZ, AMC, ATM, C, CT | 2 |
| **A7** | AMP, KF, AMC | CXM | DO, CIP, SXT, OA, CN, CTX, CAZ, ATM, C, CT | 2 |
| **A8** | AMP, KF | AMC, CXM | DO, CIP, SXT, OA, CN, CTX, CAZ, ATM, C, CT | 2 |
| **A9** | AMP, KF, OA, C | SXT | DO, CXM, CIP, CN, CTX, CAZ, AMC, ATM, CT | 2 |
| **A10** | AMP, KF, OA, C | - | DO, CXM, CIP, SXT, CN, CTX, CAZ, AMC, ATM, CT | 2 |
| **A11** | AMP, KF | CN | DO, CXM, CIP, SXT, OA, CTX, CAZ, AMC, ATM, C, CT | 2 |
| **A12** | AMP, KF, OA, C | DO, SXT | CXM, CIP, CN, CTX, CAZ, AMC, ATM, CT | 1 |
| **A13** | AMP, KF, C | CXM | DO, CIP, SXT, OA, CN, CTX, CAZ, AMC, ATM, CT | 1 |
| **A14** | AMP, KF | CXM, CN | DO, CIP, SXT, OA, CTX, CAZ, AMC, ATM, C, CT | 1 |
| **A15** | - | AMP, KF | DO, CXM, CIP, SXT, OA, CN, CTX, CAZ, AMC, ATM, C, CT | 1 |
| **A16** | AMP | - | KF, DO, CXM, CIP, SXT, OA, CN, CTX, CAZ, AMC, ATM, C, CT | 1 |
| **A17** | KF | AMP, AMC | DO, CXM, CIP, SXT, OA, CN, CTX, CAZ, ATM, C, CT | 1 |
| **A18** | - | - | AMP, KF, DO, CXM, CIP, SXT, OA, CN, CTX, CAZ, AMC, ATM, C, CT | 1 |
| **A19** | KF | - | AMP, DO, CXM, CIP, SXT, OA, CN, CTX, CAZ, AMC, ATM, C, CT | 1 |
| **A20** | AMP, KF, C | SXT | DO, CXM, CIP, OA, CN, CTX, CAZ, AMC, ATM, CT | 1 |

Isolates belonging to individual antibiograms are shown in Fig. S1.

Antibiotic abbreviations: ampicillin (AMP), cephalothin (KF), doxycycline (DO), cefuroxime (CXM), ciprofloxacin (CIP), sulfamethoxazole-trimethoprim (SXT), oxolinic acid (OA), gentamicin (CN), cefotaxime (CTX), ceftazidime (CAZ), amoxicillin with clavulanic acid (AMC), aztreonam (ATM), chloramphenicol (C), and colistin sulphate (CT)
